# The impact of splinting timepoint of mobile mandibular incisors on the outcome of periodontal treatment—preliminary observations from a randomized clinical trial

**DOI:** 10.1007/s00784-021-04075-4

**Published:** 2021-07-26

**Authors:** Sarah K. Sonnenschein, Antonio Ciardo, Samuel Kilian, Philipp Ziegler, Maurice Ruetters, Marcia Spindler, Ti-Sun Kim

**Affiliations:** 1 Section of Periodontology, Department of Conservative Dentistry, Clinic for Oral-, Dental- and Maxillofacial Diseases, University Hospital Heidelberg, Im Neuenheimer Feld 400, 69120 Heidelberg, Germany; 2 Institute of Medical Biometry and Informatics, University of Heidelberg, Heidelberg, Germany

**Keywords:** Splinting therapy, Tooth mobility, Periodontal therapy, Fiber-reinforced composite splint, Oral Health-Related Quality of Life

## Abstract

**Objectives:**

To compare the outcome of periodontal parameters in mobile mandibular incisors which were splinted before or after full-mouth disinfection (FMD).

**Materials and methods:**

Thirty-four periodontitis patients with ≥ 1 mobile mandibular incisor (mobility degree II/III, clinical attachment loss (CAL) ≥ 5 mm, relative bone loss ≥ 50%) were randomly allocated to group A or B. Patients received periodontal treatment (PT) including splinting of teeth 33–43 before (A) or after FMD (B). Patient (age/sex/smoking status/systemic diseases/number of teeth) and tooth-related parameters (mean probing pocket depth (PPD)/CAL/oral hygiene indices; for the overall dentition and region 33–43) were assessed prior to PT and 12 months after FMD by a blinded examiner. Therapy-related information was added (group/antibiotic therapy/surgical intervention).

**Results:**

Twenty-six patients (A: 12; B:14) were re-examined. Two patients of group B did not need splinting after FMD because of reduction in mobility after FMD. Regression analysis revealed a positive association of antibiotic therapy with CAL_overall, PPD_overall, and PPD_33-43 (*p* ≤ 0.01). There is a trend toward a higher reduction of periodontal parameters at teeth 33–43 in group A (PPD_33-43: − 0.91 vs. − 0.27 mm; CAL_33-43: − 1.02 vs. − 0.47 mm).

**Conclusions:**

Teeth splinted before or after FMD show a significant improvement in periodontal parameters 12 months after FMD. Splinting after FMD offered the option to detect reduction in mobility.

**Clinical relevance:**

Despite a higher, but not statistically significant, improvement in periodontal parameters on teeth splinted before FMD, the results do not indicate which timepoint of splinting is more beneficial. The decision for the therapeutic procedure should therefore be made individually.

## Introduction

The primary features of periodontitis include the loss of periodontal tissue support, clinical attachment loss (CAL) and alveolar bone loss, presence of increased periodontal pocket depth, and gingival bleeding [1]. Disease progression may lead to pathological tooth mobility which can result from acute periodontal inflammation, traumatic occlusion, and an apical shift of the rotational center of the tooth as it occurs in advanced alveolar bone loss. Patients with severe periodontitis often have a combination of these conditions, and the increased mobility can cause inconveniences for the patient. The new classification of periodontal disease states that teeth with progressive mobility may require splinting therapy to improve patient comfort [2]. Recent evidence also indicates a trend toward additional improvement for the Oral Health-Related Quality of Life (OHRQoL) of periodontitis patients by splinting mobile incisors as part of periodontal therapy [3], and retrospective studies show high survival rates and periodontal stability of splinted teeth during long-term supportive periodontal therapy (SPT) [3, 4]. However, it is unclear which timepoint during systematic periodontal treatment is optimal for splinting of mobile teeth. There is only limited evidence on this topic, and so dentists often decide according to individual preferences or due to the request of the patient. Patients affected by increased tooth mobility are often afraid of tooth loss and expect swift improvements after therapy which argues for splinting mobile teeth at the beginning of the systematic periodontal treatment. Furthermore, it is manually easier to perform subgingival debridement at non-mobile teeth. The idea that splinting reduces potential scaling-induced trauma is also widely accepted [5]. In addition, there is literature that indicates a possible influence of baseline tooth mobility on clinical outcomes of regenerative treatment of deep intrabony defects, with better outcomes at teeth with low mobility [6].

On the other hand, the elimination of periodontal inflammation and the correction of occlusal pre-contacts can favor regeneration of the surrounding tissues and thereby reduce tooth mobility. Furthermore, changes in tooth position caused by swelling are also reversible. From this point of view, there is also a strong rationale for splinting mobile teeth after active periodontal treatment.

Hence, the aim of the study is to evaluate the impact of splinting periodontally compromised mobile mandibular incisors with unfavorable prognosis on the Oral Health-Related Quality of Life (OHRQoL) and on the change of periodontal parameters on the splinted teeth after periodontal therapy in a prospective and randomized study design over a period of 5 years. The results presented are short-term results 12 months after FMD. This is the second publication in the scope of the study.

## Materials and methods

The study participants were recruited between November 2016 and December 2018 from patients of the authors’ department. Inclusion criteria at the patient level were presence of periodontitis with at least 6 teeth with a probing pocket depths (PPD) ≥ 4 mm, age ≥ 18 years, and presence of ≥ 12 natural teeth. Tooth-related inclusion criteria were presence of at least one mandibular incisor with a mobility degree II or III [7] in combination with a clinical attachment loss (CAL) ≥ 5 mm and a relative alveolar bone loss (ABL) of ≥ 50% at the affected tooth. Patients with a cross or head bite, stress-induced bruxism, an implant in the mandibular anterior region, or active periodontal therapy (APT) within the last 2 years were excluded from the study. Primary outcome variables were mean CAL and mean PPD of teeth 33 to 43 before systematic periodontal treatment (baseline, BL) and 12 months after FMD (T2). The randomization of the included patients was performed via selected envelopes using block randomization with a 1:1 ratio for the assignment to group A or group B. Patients of group A received splinting of teeth 33 to 43 prior to full-mouth disinfection (FMD) and group B 7 months after FMD.

### Active periodontal therapy

**Fig. 1:**
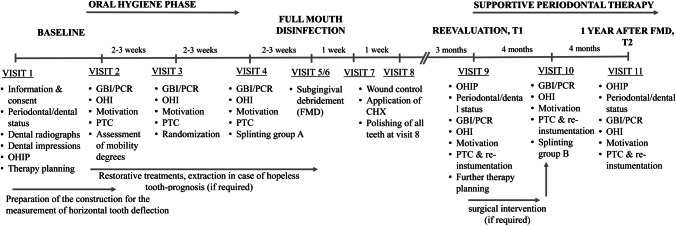
Flowchart of the study design. CHX, chlorhexidine gel 1%; FMD, full-mouth disinfection; GBI, gingival bleeding index (Ainamo & Bay, 1975); OHI, oral hygiene instructions; OHIP, Oral Health Impact Profile G-14 questionnaire; PCR, plaque control record (O'Leary, Drake, & Naylor, 1972); PTC, professional tooth cleaning

APT of all patients was performed according to the department’s concept of systematic periodontal therapy (PT). This meant a total of nine sessions (visit 1 to visit 9) for each study participant during the oral hygiene phase and non-surgical periodontal therapy. BL periodontal status and the medical history (including smoking status and presence of systemic diseases) were assessed at visit 1. BL oral hygiene indices were assessed at visit 2. Non-surgical periodontal therapy (performed by a dental hygienist) included the removal of all subgingival deposits at visits 5/6 according to a modified concept of full-mouth disinfection (FMD) [8] as described previously [3] and the adjustment of occlusion in case of premature contacts. If necessary, an adjunctive antibiotic administration (500-mg amoxicillin and 400-mg metronidazole, three times per day for 7 days) was performed according to the current recommendations of the German Society for Periodontology[9, 10].^1^ The outcome of non-surgical periodontal therapy was re-evaluated 3 months after FMD (visit 9). Remaining pockets of 4 mm and bleeding on probing (BOP) and pockets of 5 mm were re-instrumented at this visit and at following supportive periodontal therapy (SPT) sessions. Patients with remaining sites ≥ 6 mm and/or furcation involvement were recommended to undergo further surgical interventions (visit 9b). Mandibular incisors and canines were excluded from additional surgical treatment. After completion of APT, patients were referred to SPT every 4 months (visits 10 and 11). The oral hygiene indices recorded at all sessions of the oral hygiene phase and SPT were the plaque control record (PCR) [11] and the gingival bleeding index (GBI) [12]. Patients were scheduled for re-examination 12 months after FMD and completion of APT by a blinded examiner (T2; 12 months ± 8 weeks after FMD). The procedure of the study is shown in Fig. 1.

### Assessment of periodontal parameters and mobility degrees

Periodontal status was assessed with PPD and CAL measured at 6 sites/tooth (PCP-UNC15 probe, Hu-Friedy, Frankfurt, Germany). Except for one patient, all periodontal status at BL were assessed and documented (ParoStatus®, ParoStatus.de, Berlin, Germany) by one of two calibrated examiners. At T2, patients were followed up by an examiner who was blinded to the group affiliation. Measured against a reference model, the relative agreement of all examiners (measurement accuracy of ± 1 mm) was 89.3–96.0% for the CAL and 94.6–99.3% for the PPD.

The horizontal deflection of the mandibular incisors was measured in millimeters using a new method as described previously [3]. All measurements were performed by the same examiner and then converted to a modified Lindhe and Nyman degree classification [7] (degree I: pathological mobility ≤ 1 mm in labio-oral direction, degree II: mobility of > 1–2 mm, degree III: exceeding 2 mm in labial-oral direction and/or in vertical direction).

### Assessment of Oral Health-Related Quality of Life

The patients’ Oral Health-Related Quality of Life (OHRQoL) was assessed using the German short version of the Oral Health Impact Profile (OHIP-G14) [13]. The OHIP-G14 questionnaires were self-completed by the participants at BL and T2. Responses of the OHIP-G14 are summed to give the total OHIP-G14 summary score and can range from 0 to 56 with a high score indicating a poorer OHRQoL.

### Splinting

**Fig. 2:**
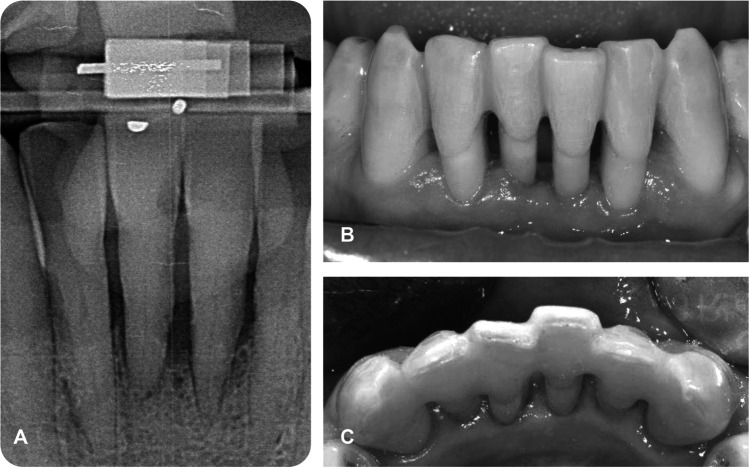
Standardized radiograph (**A**) of the mandibular front of a study participant prior to periodontal therapy (baseline) and clinical situation (**B**, **C**) 12 months after full-mouth disinfection and splinting of teeth 33 to 43 (T2)

Patients of group A received splinting of mobile mandibular incisors prior to FMD (visit 4), while patients of group B received splinting therapy 7 months after FMD (visit 10). In all patients, teeth 33 to 43 were splinted using composite (Tetric EvoCeram/Flow, IvoclarVivadent, Ellwangen, Germany) and a fiber-reinforced composite (FRC) strand (everStick Perio, GC Germany, Bad Homburg, Germany) (Fig. 2). Canines were included into splints for stability. The mobility of a canine was not an exclusion criterion. Even in the case of mobility of a canine, the splinting was inserted only from 33 to 43. All splints were inserted by the same dentist according to a standardized protocol as described previously [3]. The adjustment of occlusion in case of premature contacts of teeth 33–43 was performed, if necessary. Patients were instructed how to clean the splinted teeth (adaption and handling of interdental brushes).

### Statistical analysis

Statistical analysis was done based on the set of patients who completed follow-up at T2. As this study is explorative, no formal sample size calculation was performed. The sample size of 34 was chosen based on considerations of feasibility and was considered sufficient to obtain first estimates of group differences regarding the various variables. The recorded periodontal status data was exported from the documentation software (ParoStatus®, ParoStatus.de, Berlin, Germany) to a table calculation program (Excel®, Microsoft). All other data and the oral hygiene indices were entered manually into the same tabular program independently by two different persons. Any discrepancies were corrected accordingly after the original documents were reviewed again. Descriptive statistics for periodontal parameters and oral hygiene parameters were assessed by calculating means, standard deviation, median, first and third quantile, minimum, and maximum. BL values and BL-T2 differences were compared between the two groups using the chi-square test for binary variables and the Mann–Whitney U test for all other variables. Corresponding 95% confidence intervals (CI) for the proportion difference (binary variables) or the median of differences (other variables) are given. The patient was considered as a statistical unit. The two timepoints BL and T2 are compared within groups using the Wilcoxon two-sample signed-rank test. Corresponding 95% confidence intervals of the median of differences are given. A possible association of the periodontal situation (mean CAL_overall, mean CAL_33-43, mean PPD_overall, mean PPD_33-43) and possible influencing factors was analyzed using multivariate regression with covariates (group [A/B], smoking status [non-smoker/smoker], antibiotic therapy [not received/received], systemic factors [not present/present], surgical intervention [not received/received]). All *p*-values are to be interpreted descriptively; thus, no adjustment for multiple testing was performed. *p*-values below 0.05 were regarded as considerable. Third molars and dental implants were excluded from analysis. Analysis was done using the statistical software R v. 4.0.1 (The R Project, The R Foundation).

## Results

A total of 34 patients met the inclusion criteria and agreed to participate. Until T2, a total of eight patients dropped out: Two patients because of health reasons, five patients discontinued therapy for unknown reasons, and one patient withdrew his consent. Accordingly, 26 study participants could be included in the statistical analysis (group A: 12 patients, group B: 14 patients). Two patients were re-examined (T2) 2 to 3 months later than planned due to SARS-CoV-2 pandemic. Thus, re-examinations at T2 took place between February 2018 and August 2020.

### Descriptive statistic

Descriptive statistic of the study cohort is shown in Table 1.

**Table 1:** Descriptive characteristics of the study groups

Variables	Group A (*n* = 12)	Group B (*n* = 14)	*p*-value	95% CI
Sex				
Male	4 (33%)	5 (36%)	> 0.999^chi2^	[− 0.45, 0.4]^PrDiff^
Female	8 (67%)	9 (64%)		
Age at BL				
Mean	55	55	0.877^MWU^	[− 9, 9]^MedDiff^
sd	11	9.3		
Median	56	55		
Q1–Q3	46—64	53–58		
Min–max	37–68	32–70		
Number of teeth at BL				
Mean	21	23	0.327^MWU^	[− 7, 2]^MedDiff^
sd	5.3	4		
Median	22	24		
Q1–Q3	16—24	22—26		
Min–max	12—28	14—28		
Number of teeth at T2				
Mean	20	22	0.380^MWU^	[− 6, 2]^MedDiff^
sd	5.2	4.2		
Median	22	22		
Q1–Q3	15—24	18—25		
Min–max	12—27	14—28		
Smoking status				
Non-smokers	6 (50%)†	9 (64%)	0.736^chi2^	[− 0.61, 0.32]^PrDiff^
Smokers	6 (50%)	5 (36%)		
Diabetes mellitus				
Type I	1 (8%)‡	0		
Type II	0	4 (29%)‡		
Systemic factors §				
Not present	10 (83%)	9 (64%)	0.517^chi2^	[− 0.26, 0.74]^PrDiff^
Present	2 (17%)	5 (36%)		
Adjunctive antibiotic therapy				
Not received	8 (67%)	8 (57%)	0.926^chi2^	[− 0.37, 0.57]^PrDiff^
Received	4 (33%)	6 (43%)		
Surgical intervention				
Not received	3 (25%)	8 (57%)	0.209^chi2^	[− 0.77, 0.11]^PrDiff^
Received	9 (75%)	6 (43%)		
Periodontal diagnosis				
Stage IV grade C	6 (50%)	7 (50%)		
Stage III grade C	6 (50%)	6 (43%)		
Stage III grade B	0	1 (7%)		
Distribution of mobility degrees of mandibular incisors¶				
No pathological mobility	3 teeth (6.7%)	4 teeth (7.1%)		
Mobility degree I	20 teeth (44.4%)	32 teeth (57.1%)		
Mobility degree II	15 teeth (33.3%)	12 teeth (21.4%)		
Mobility degree III	7 teeth (15.6%)	8 teeth (14.3%)		

*chi2*, chi-square test; *MWU*, Mann–Whitney U test; *PrDiff*, CI for the difference of proportions; *MedDiff*, CI for median of differences; †, including 2 former smokers (defined as patients that stopped smoking ≥ 5 years ago); ‡, HbA1c ≥ 7.0% in all patients; §, systemic diseases/factors were defined as the present of one or more of following diseases in a patient, rheumatoid arthritis, history of tumor disease (only included in case of treatment with chemotherapeutics or medications with impact on the bone metabolism), osteoporosis, or diabetes mellitus; ¶, mobility degrees of mandibular incisors (32, 31, 41, 42) at BL (according to Lindhe & Nyman, teeth intended for extraction excluded); BL, baseline (= prior to periodontal treatment); T2, 12 months after full-mouth disinfection

### Survival of splinted teeth and splints

At BL, all patients except one had all mandibular incisors and canines. In one patient of group A, one incisor was missing due to aplasia of the tooth and gap closure was present. Thus, at BL a total of 155 teeth were present in the area of teeth 33–43 (group A: 71 teeth, group B: 84 teeth). Due to the hopeless prognosis (according to Kwok and Caton [14]), two patients of group A underwent removal of one incisor prior to splinting therapy. Accordingly, in three patients of group A, one tooth was missing in the mandibular anterior region, and only five teeth were splinted together at visit 4. In the two extraction cases, the missing tooth was replaced with an adhesively fixed pontic. The distribution of BL mobility of the mandibular incisors is given in Table 1. In two patients of group B, the mobility decreased so much until visit 10 that splinting was no longer indicated (mobility degree decreased from II and III to 0 and I). At T2, none of teeth 33 to 43 were lost. In one patient of group A, debonding of an incisor from the splint occurred 12 months after splinting. The affected splint was fixed prior to blinded re-examination at T2. No other complications or fractures of the splints were observed.

### Periodontal parameters and oral hygiene indices

**Fig. 3:**
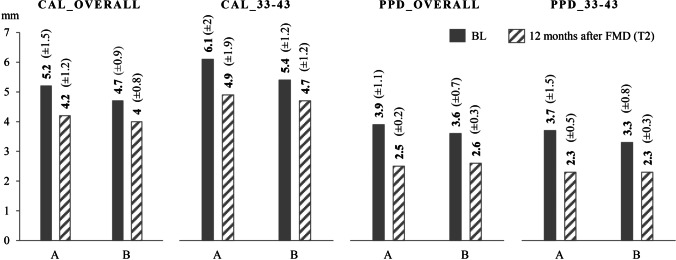
Mean values of the periodontal status at baseline (BL) and 12 months after full-mouth disinfection (T2). CAL_overall, mean clinical attachment level of the overall dentition (including all teeth); CAL_33-43, mean clinical attachment level of teeth 33 to 43; PPD_overall, mean probing pocket depth of the overall dentition (including all teeth); PPD_33-43, mean probing pocket depth of teeth 33 to 43; A, group A (*n* = 12, splinting therapy prior to FMD); B, group B (*n* = 14, splinting therapy 7 months after FMD)

**Fig. 4:**
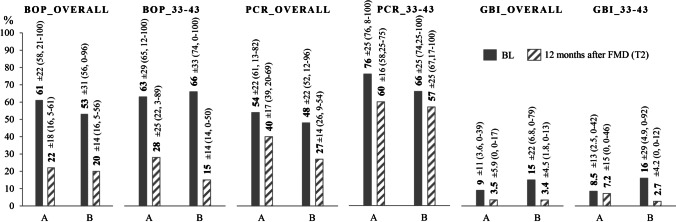
Mean values (± standard deviation, median, minimum–maximum) of the oral hygiene indices and bleeding on probing at baseline (BL) and 12 months after full-mouth disinfection (T2). BOP_overall, mean bleeding on probing of the overall dentition (including all teeth); BOP_33-43, mean bleeding on probing of teeth 33 to 43; PCR_overall, mean plaque control record (O'Leary, Drake, & Naylor, 1972) of the overall dentition (including all teeth); PCR_33-43, mean plaque control record (O'Leary, Drake, & Naylor, 1972) of teeth 33 to 43; GBI_overall, mean gingival bleeding index (Ainamo & Bay, 1975) of the overall dentition (including all teeth); GBI_33-43, mean gingival bleeding index (Ainamo & Bay, 1975) of teeth 33 to 43; A, group A (*n* = 12, splinting therapy prior to FMD); B, group B (*n* = 14, splinting therapy 7 months after FMD)

**Fig. 5:**
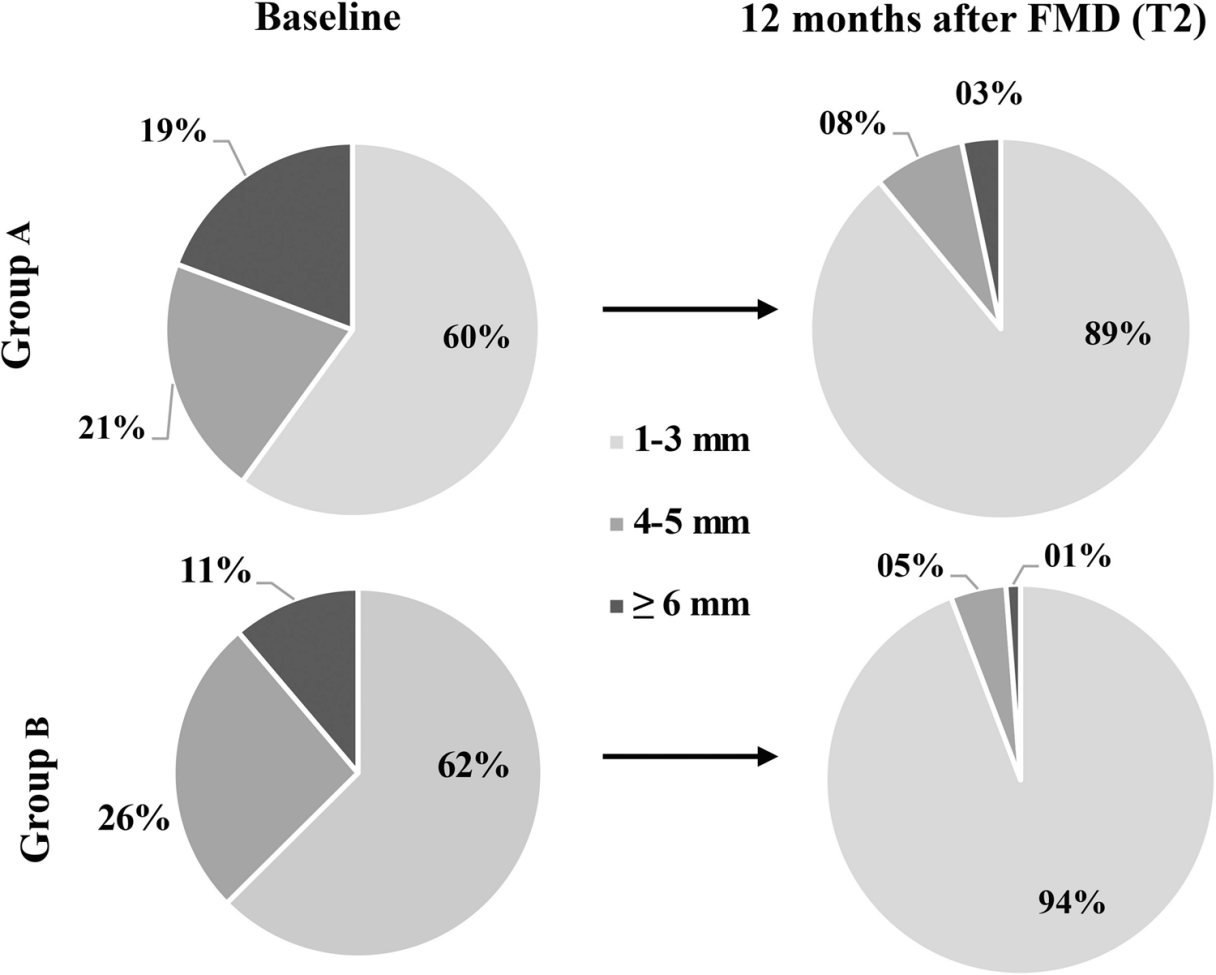
Distribution of probing pocket depths at teeth 33 to 43. Group A: 71 teeth (426 probing sites) at baseline and 69 teeth (412 probing sites) at T2. Group B: 84 teeth (504 probing sites) at baseline and T2. FMD, full-mouth disinfection; T2, 12 months after FMD

The mean values of the periodontal status and oral hygiene indices at both examination points are shown in Figs. 3 and 4. The distribution of PPD at teeth 33 to 43 is shown in Fig. 5. Group differences at the examination points are not found either for periodontal parameters or oral hygiene indices. However, the PCR_overall at T2 tends to show a difference (*p* = 0.060). In both groups, the mean CAL and PPD of the overall dentition and at teeth 33–43 improved significantly from BL to T2 (al p ≤ 0.005).

Regression analyses of CAL and PPD changes (from BL to T2) for the overall dentition and the local changes at teeth 33–43 show a positive association with adjunctive antibiotic administration for PPD_33-43, PPD_overall, and CAL_overall. For the changes in area 33–43, the regression analysis also shows a tendency toward a higher reduction of periodontal parameters within group A compared to group B (PPD_33-43: − 0.91 vs. − 0.27 mm; CAL_33-43: − 1.02 vs. − 0.47 mm) (Table 2).

**Table 2:** Regression analysis of changes in periodontal parameters with potential influencing factors for the therapy outcome

	Estimate	Std. error	*p*-value	
Δ CAL_overall				
(Intercept)	− 0.65	0.21	0.005	**
Group B	0.34	0.18	0.079	
Smoking status	− 0.15	0.17	0.394	
Systemic factors	0.07	0.16	0.675	
Antibiotics	− 0.60	0.18	0.003	**
Surgical intervention	− 0.02	0.18	0.902	
Δ CAL_33-43				
(Intercept)	− 1.02	0.36	0.009	**
Group B	0.55	0.38	0.169	
Smoking status	− 0.18	0.39	0.643	
Systemic factors	− 0.25	0.35	0.491	
Antibiotics	− 0.18	0.39	0.643	
Δ PPD_overall				
(Intercept)	− 0.76	0.26	0.009	**
Group B	0.45	0.23	0.067	
Smoking status	0.12	0.22	0.605	
Systemic factors	− 0.28	0.20	0.180	
Antibiotics	− 1.16	0.22	< 0.001	***
Surgical intervention	− 0.28	0.23	0.234	
Δ PPD_33-43				
(Intercept)	− 0.91	0.36	0.019	*
Group B	0.64	0.39	0.112	
Smoking status	− 0.02	0.39	0.960	
Systemic factors	− 0.48	0.35	0.184	
Antibiotics	− 1.26	0.39	0.004	**

*Δ CAL_overall*, change of the baseline mean clinical attachment level (CAL) of the overall dentition from baseline (BL) to 12 months after full-mouth disinfection (= T2-BL); *Δ CAL_33-43*, change of the baseline mean clinical attachment level (CAL) of teeth 33 to 43 from BL to T2 (= T2-BL); *Δ PPD_overall*, change of the baseline mean probing pocket depth (PPD) of the overall dentition from BL to T2 (= T2-BL); *Δ PPD_33-43*, change of the baseline mean probing pocket depth (PPD) of teeth 33 to 43 from BL to T2 (= T2-BL);., *p* < 0.07; *, *p* ≤ 0.05; **, *p* ≤ 0.01; ***, *p* < 0.001

### Oral Health-Related Quality of Life

At T2, the mean OHIP-G14 summary score of the entire study population is 10.7 ± 7.7 (median: 8.5; range: 0–25). For group A, the mean OHIP-G14 score is 10.5 ± 6.8 (median: 10; range: 0–18) and for group B 10.7 ± 7.7 (median: 8.5, range: 0–25).

## Discussion

This study prospectively investigates the 12-month outcome of PT at mobile mandibular incisors which were splinted from canine to canine either prior to FMD or 7 months after FMD. The periodontal situation was significantly improved by PT. Patients who received adjunctive antibiotic therapy showed a higher reduction of the overall CAL and PPD and the PPD at teeth 33 to 43. That adjunctive antibiotic therapy leads to better therapy outcomes, especially in the reduction and proportion of PPD from initial deep pockets ≥ 5 mm, has already been demonstrated [15, 16]. In the present study, the proportion of deep pockets was relatively high at teeth 33–43, which may explain the better outcome in this area in patients with adjunctive antibiotic administration. For the patients who received splinting after FMD, there was a tendency for a smaller reduction of the overall CAL and PPD. This might also be due to the higher proportion of diabetics in this group in which the outcome of periodontal therapy can be negatively affected by the systemic conditions [17]. Furthermore, the initial periodontal situation in group B was better compared to that in group A. At T2, both groups were then at a similar periodontal level. Thus, the improvement in group A was higher than that in group B. This difference could also be caused by the “regression to the mean” effect.

In two patients who should have received splinting therapy after FMD, splinting was no longer indicated due to a significant decrease in mobility. None splinted tooth got lost during the observation period and only one splint showed debonding of a single tooth. Thus, high survival rates were observed for both the splinted teeth and the splints. Although there are only a few other prospective studies investigating the survival rates of splinted teeth and splints, the results are quite different. In accordance with our results, Kumbuloglu et al. [18] also found a remarkably high survival rate for splints in their prospective observation of 19 periodontitis patients that had splinting therapy with FRC strands and composite from mandibular canine to canine. After 4.5 years, the survival rate of splints was 94.8%, and none of the splinted teeth was lost during the observation period. In contrast, Sekhar et al. [19] observed relatively many splint fractures in their prospective study. During a period of 12 weeks, eleven out of 20 splints showed fractures.

In current literature on splinting therapy of periodontally compromised and mobile teeth, retrospective studies present the largest patient cohorts. Here, remarkably high survival rates of splinted teeth were also observed over periods of 11 [4] up to 12 years [20]. Thus, Graetz et al. [4] found that splinted teeth were not at higher risk for tooth loss compared to non-splinted teeth. They included 57 patients with 227 splinted teeth over a mean observation period of 11 years in their analysis. Only 26 splinted teeth were lost during the mean observation time but 75.3% of all splints required repair. It should be noted that all types of teeth were included in this study and that splints on lower anterior teeth required fewer repairs, while repairs tended to be more likely in posterior teeth. Sonnenschein et al. [20] also observed no tooth loss in 39 patients with 162 splinted mandibular anterior teeth within the first 3 years after splint placement in a retrospective study. After 7 (24 patients, 98 splinted teeth) and 12 years (16 patients, 71 splinted teeth), one splinted tooth was lost in each case. In contrast to the study by Graetz et al. [4], this study found a high survival rate of splints. A total of 74.4% of the original splints were still intact after 3 years and 67.3% after 10 years.

The discussed studies do not address the question of whether splinting is more beneficial before or after subgingival instrumentation but Alkan et al. [5] investigated this question. They examined ten patients who received splinting of mandibular incisors before non-surgical subgingival debridement and eleven patients who received splinting therapy after the subgingival debridement. There were no differences in the outcome of periodontal therapy after 6 months, and the authors conclude that splinting of periodontally compromised teeth prior to non-surgical subgingival debridement and thus the elimination of potential scaling-induced trauma have no additional effect on the outcome of PT. In this study, however, the primary intention of splinting was not to eliminate tooth mobility to improve oral comfort and the patient’s chewing and biting function but rather to determine whether immobilization by splinting provides better healing and thus a better therapeutic outcome. In contrast, other studies indicate a possible influence of baseline tooth mobility on clinical outcomes of regenerative treatment, with better outcomes at teeth with low mobility [6]. In the presented study, the patients who received splinting of teeth 33 to 43 before FMD showed a tendency toward a better outcome of PT in the splinting area. A possible explanation for this is better healing due to stabilization of teeth preventing early disruption of the blood clot from the root surface. Thus, the timepoint at which mobile teeth are splinted during systematic periodontal treatment could potentially have an impact on the therapy outcome.

A common intention of splinting therapy is to improve the oral comfort of patients affected by severe tooth mobility. The 3-month results of the presented study population investigated the impact of splinting on OHRQoL and found a trend toward better OHRQoL in patients who had additional splinting therapy compared to the non-splinted control group [3]. Twelve months after FMD (both groups received splinting), the mean OHIP-G14 summary score and thus the OHRQoL are almost identical in both groups. It can therefore be assumed that OHRQoL improves more quickly with earlier splinting but that after a short time there is no difference compared to patients who received splinting later.

As already shown 3 months after FMD, high plaque scores are also found after 12 months, especially on splinted teeth. The question therefore remains whether splinting leads to a reduction of oral hygiene control at home. The increased plaque scores are also reflected in a high number of sites with BOP. Therefore, gingivitis is still observed in many patients despite the significant decrease in PPD. The follow-up will show whether this situation will improve during further SPT.

The strength of the study is its prospective and randomized design with blinded re-examination. Possible limitations of the study are the small sample size and the different distribution of diabetes between the groups. Furthermore, the different initial periodontal situation could have an influence on the results.

Future studies can use the preliminary results for sample size calculation. For example, one might choose CAL_33-43 at 12 months after FMD as primary endpoint that is analyzed with an ANCOVA adjusted for BL CAL_33-43. The observed group difference was 0.55 mm (Table 2) and the standard deviation not higher than 2 mm (Fig. 3). Employing a correlation of BL and 12-month value of 0.8 (supported by the data), significance level of 0.05, and power of 0.8, the required sample size of an ANCOVA analysis would be 76 patients per group. However, a greater group difference might be seen after a longer follow-up period.

In summary, the presented study found a tendency for better outcomes of periodontal parameters after systematic periodontal treatment when splinting mobile mandibular incisors before FMD compared to splinting 7 months after FMD. The study also shows that splinting after FMD enables to detect remission of tooth mobility and thus the opportunity to avoid splinting.

Based on the results of the study, it is not possible to state at which point systematic periodontal therapy splinting of mobile teeth is more beneficial. On the one hand, there seems to be a tendency toward a higher reduction of periodontal parameters when splinting prior to FMD and a faster improvement of the OHRQoL. On the other hand, a wait-and-see approach enables detection of remission of tooth mobility. Independent of the timepoint of splinting therapy, it seems that more intense oral hygiene instructions and short SPT intervals are required to ensure the shortcomings from limited personal oral hygiene efficiency due to splinting. Future research will show how periodontal parameters, survival rate of splinted teeth, and splints will develop in the long-term and whether recommendations for the timepoint of splinting can be derived on this basis.

## Data Availability

The data that support the findings of this study are available from the corresponding author upon reasonable request.

## Footnotes

1 Guidelines of the German Society for Periodontology (Deutsche Gesellschaft für Parodontologie, DGPARO) changed during the study period (year 2018). The indication for adjunctive antibiotic administration was made according to the respective recommendations.
