# Correction to: The impact of splinting timepoint of mobile mandibular incisors on the outcome of periodontal treatment—preliminary observations from a randomized clinical trial

**DOI:** 10.1007/s00784-022-04654-z

**Published:** 2022-07-28

**Authors:** Sarah K. Sonnenschein, Antonio Ciardo, Samuel Kilian, Philipp Ziegler, Maurice Ruetters, Marcia Splindler, Ti‑Sun Kim

**Affiliations:** 1 Section of Periodontology, Department of Conservative Dentistry, Clinic for Oral-, Dental- and Maxillofacial Diseases, University Hospital Heidelberg, Im Neuenheimer Feld 400, 69120 Heidelberg, Germany; 2 Institute of Medical Biometry and Informatics, University of Heidelberg, Heidelberg, Germany

**Correction to: Clinical Oral Investigations**

**https://doi.org/10.1007/s00784-021-04075-4**

In the published paper, the last name of the author Marcia Spindler was incorrectly written as Marcia Splindler.

The original article has been corrected.

